# Non-toxigenic cases of Vibrio cholerae in Spain from 2012 to 2022

**DOI:** 10.1099/mgen.0.001315

**Published:** 2024-12-11

**Authors:** Camille Jacqueline, Sergio Román Soto, Silvia Herrera-Leon

**Affiliations:** 1 Centro Nacional de Microbiología, Instituto de Salud Carlos III, Majadahonda, Spain; 2 European Public Health Microbiology Training Program (EUPHEM), European Centre for Disease Prevention and Control (ECDC), Stockholm, Sweden; 3 Laboratorio de Microbiología Clínica y Biología Molecular, Hospital Comarcal de Melilla, Rusadir, Spain

**Keywords:** antimicrobial resistance, hospitalization, logistic regression, surveillance, whole-genome sequencing

## Abstract

Non-toxigenic non-O1/non-O139 *Vibrio cholerae* (NVC) isolates are associated with diarrhoeal disease globally. NVC-related infections are on the rise, representing one of the most striking examples of emerging human diseases linked to climate change. This study aims to give a better picture of the evolution of NCV incidence in Spain from 2012 to 2022. In this context, we realized a descriptive analysis and a logistic regression using the isolates submitted to the National Center of Microbiology (NCM) during this period. To elucidate the heterogeneity of sporadic clinical strains of NVC among patients residing in Spain, we conducted whole-genome sequencing (WGS) of a selection of isolates. First, we observed an increase in the number of isolates sent to the NCM after 2019, which was not concomitant to a change in the national surveillance protocol. Furthermore, the number of cases and hospitalizations increased with age. Second, we found a high diversity of NVC strains, which suggested that the usefulness of WGS studies might be limited in waterborne outbreak situations to find the infectious source. Finally, we characterized the genetic determinants responsible for antimicrobial resistance and virulence and found that 21% of the isolates were resistant to *β*-lactamases. To the best of our knowledge, the present study is the first in Spain to report genomic data on non-toxigenic cases at the national level. Because of the high percentage of hospitalization observed for NVC cases (40%), it might be beneficial to test for *V. cholerae* in all the suspected cases.

**Impact Statement**

The main concerns regarding non-toxigenic non-O1 and non-O139 *Vibrio cholerae* (NVC) isolates are (i) whether they can gain toxin genes and cause epidemic cholera and (ii) that they cause extraintestinal diseases that are on some occasions life-threatening (e.g. septicaemia, necrotizing fasciitis, etc.). Most European countries do not routinely check for the presence of NVC in clinical samples, and therefore, very few studies are available on the epidemiology and phenotypic and genetic characteristics of the NVC isolates. In this study, we report an increase in the number of NVC isolates sent to the national reference centre since 2019 as well as an increase in the probability of being infected and hospitalized with the age of the case. The findings also suggest that epidemiological studies are crucial to identifying the infection source as whole-genome sequencing (WGS) studies are limited by the high diversity of NCV in water samples. These findings contribute valuable insights into the circulation of NCV in Spain, as it is the first of this kind to be conducted at the national level in this country. Furthermore, it highlights the need to strengthen the surveillance of NVC cases to get a better understanding of the incidence of the disease and of the risk factors for severity.

## Data Summary

All NVC isolate whole-genome sequences used in this work are available under Bioproject PRJNA1081302. Strain-specific information can be found in Table S1, available in the online version of this article.

The authors confirm that all supporting data, code and protocols have been provided within the article or through supplementary data files. Three supplementary files are available with the online version of this article.

## Introduction

*Vibrio cholerae* is the causative agent of cholera, an acutely dehydrating diarrhoeal disease that can kill its victims within hours if left untreated. In contrast to *V. cholerae* serotype O1 and O139, the non-toxigenic non-O1/non-O139 *V. cholerae* (NVC) is not associated with cholera epidemics but with sporadic cases or small outbreaks of gastrointestinal diseases. Occasionally, however, these can cause extraintestinal diseases that may range in severity from mild (e.g. otitis) to life-threatening (e.g. septicaemia, necrotic fasciitis, etc.) [1]. NVC is part of the normal bacterial salt and freshwater ecosystem, and cases can occur either by ingestion of contaminated seafood, drinking water consumption or during recreational activities [2]. Although generally neglected, NVC-related infections are on the rise and represent one of the most striking examples of emerging human diseases linked to climate change. Indeed, elevated numbers of NVC‐related infections were reported in Europe, especially in the countries bordering the Baltic Sea during summer heat waves [3,5], and also in the Netherlands [6,7], Belgium [8], France [9], Croatia [10], Austria [11] and Slovakia [12].

Most European countries do not routinely check for the presence of NVC in clinical samples and therefore very few studies are available on the genetic diversity as well as on the phenotypic and genetic characteristics of the NVC isolates. This is an important gap in public health action, as NVC is known to carry a number of virulence factors, also found in the toxigenic strains, which contribute to the infection process [13,14]. In 2019, a German study revealed a considerable heterogeneity of sequence types in the environmental and clinical isolates [15]. In addition, it showed that the environmental strains shared many of the virulence factors harboured by the clinical strains. Recently, NVC was also isolated for the first time in natural and artificial lakes and ponds in Serbia and the authors showed that the strains harboured pathogenicity factors such as *hlyA*, *toxR* and *ompU* [16]. While only eight imported cases of toxigenic (O1 and O139) *V. cholerae* were reported in Spain since 2015, domestic cases of NVC are frequent and their characteristics have not yet been described. In this study, we aimed to fill this knowledge gap by describing the evolution of the number of NVC cases reported since 2012 and by studying the phenotypic and genetic characteristics of a selection of environmental and clinical isolates. In addition, we explored the added value of genomic methods during the investigation of NVC outbreaks related to water used for human consumption.

## Methods

### Case definition

For this investigation, we defined a case as a patient with a laboratory-confirmed *V. cholerae* non-O1/non-O139 infection for which a specimen was collected between 1 January 2012 and 31 December 2022. Travel-associated cases were excluded.

### Isolate collection

Over the study period, hospitals voluntarily sent 92 NVC isolates to the National Center of Microbiology (NCM), and the epidemiological data were collected through laboratory request forms filled by the hospitals when sending isolates to the NCM. After reception, all isolates underwent experimental serotyping based on the O antigen by slide agglutination. Whole-genome sequencing (WGS) is not systematically performed for non-toxigenic *V. cholerae* in Spain. To study the potential of genomic surveillance in the cases of NVC, we selected a subset of 27 sequences from 2019 to 2022 including 15 sporadic cases and 2 waterborne events. They included human and environmental samples and were representative of the geographical and temporal distribution of the waterborne events (Table S1).

### Epidemiological data and statistical analyses

The following data were collected for each case: date of sampling, date of birth and age, sex, region of residence, hospitalization (yes/no), outcome (alive, dead or unknown), species and serogroup of the bacterial agent responsible for the infection. Data from the cases included in the genomic analyses are presented in Table S1. We used a linear regression model to study the evolution of the number of cases received at the NCM. *χ*^2^ tests were used to compare the probability of being a case across sexes and age groups. Six age groups were considered, 0–15 (*n*=8), 16–30 (*n*=3), 31–45 (*n*=12), 46–60 (*n*=17), 61–75 (*n*=18) and more than 75 years old (*n*=21). A binomial statistical model was used to study the correlation between age and hospitalization. Statistical significance was set at *P*<0.05. Statistical analyses and visualization were performed in R v3.6.1.

### Antimicrobial susceptibility testing

Susceptibility to antimicrobials was determined with disc diffusion assays using Mueller–Hinton agar and commercially available discs (Oxoid™). Testing was carried out for ampicillin, cefotaxime, ciprofloxacin, nalidixic acid, tetracycline and chloramphenicol. Isolates were designated as resistant (R), increased exposure (I) or susceptible (S) based on the available clinical breakpoint data published by the EUCAST (v13.1). For antimicrobials in which such breakpoints were not available, we used CLSI guidelines [17].

### Whole-genome sequencing

Genomic DNA was purified with the Maxwell® RSC Cultured Cells DNA Kit (Promega, Wisconsin, EUA). DNAs were quantified using a Qubit fluorometer (Invitrogen/Life Technologies, Massachusetts, EUA) before library preparation. Libraries were prepared by using Nextera XT DNA Library Preparation Kit (Illumina®, California, EUA) and cluster generation followed by paired-end sequencing (2×250 bp or 2×150 bp) was performed on NextSeq 550 instrument (Illumina®, California, EUA), according to the manufacturer’s instructions.

### Genome assembly and resistome analysis

Raw reads were uploaded on the EnteroBase online platform for *V. cholerae* (https://enterobase.warwick.ac.uk/). Quality control, trimming and assembly were performed by using the EnteroBase QAssembly pipeline v3.61 available at the EnteroBase online platform. Assemblies were screened for the presence of resistance genes (RGI from CARD, https://card. mcmaster.ca/analyze/rgi), virulence factors (VFDB, http://www.mgc.ac.cn/cgi-bin/VFs/v5/main.cgi) and prophages (PHASTER, https://phaster.ca/). Plasmid replicons were identified using PlasmidFinder v2.1 [18]. A 99% threshold for identity and a threshold of 100% for coverage were used as cut-offs for confirming a specific replicon present or absent.

### Multilocus sequence typing

Ribosomal multilocus sequence typing (rMLST) analysis was performed using 63 loci in Enterobase and PubMLST. The new ribosomal sequence types (rST) identified were submitted to PubMLST (Submission ID: BIGSdb_20240827101938 _589472_32256). We assigned a sequence type (ST) to isolates of non-toxigenic *V. cholerae* using PubMLST scheme based on seven alleles, *adk, gyrB, mdh, metE, pntA, purM* and *pyrC*, and new ST were assigned by PubMLST (see Table S2). cgMLST analysis was performed using cgMLST v2 scheme comprising 1128 target loci from Enterobase. A GrapeTree was used to build a minimum spanning tree based on the above cgMLST scheme. Isolates with less than five allelic differences (AD) were aggregated to identify clusters.

### Phylogenetic analysis

Core genome SNPs (cgSNPs) were called using Snippy v4.4.5 (https://github.com/tseemann/snippy). Sequences were aligned to genome LT907989 (chromosome 1) and LT907990 (chromosome 2). The length of the alignment was 3 082 510 bp with 142 911 parsimony-informative sites. We did not identify a significant number of SNPs in recombination regions when using Gubbins v.3.3.1 (https://github.com/nickjcroucher/gubbins) (see Table S3 for distance matrix of non-recombination regions). The obtained alignments were analysed to build a maximum-likelihood phylogenetic tree using IQ-TREE v2.1.4, in which the K3P(u)+F+I was identified as the best substitution model and was used for SNP-only alignments, using the ultrafast bootstrap option (1000 replicates) [19,20]. The tree was visualized in iTOL [21].

## Results

### Epidemiology of NCV infections

Over the 92 cases received during the study period, the NCM mostly received isolated strains (94%) from voluntary hospitals. The matrices, from which the strain was isolated, were indicated in 17 cases: 11 strains were isolated from haemocultures, 5 from ear exudates and 1 from a wound. Occasionally, faecal samples were sent and were isolated directly at the NCM (5%).

**Fig. 1.**
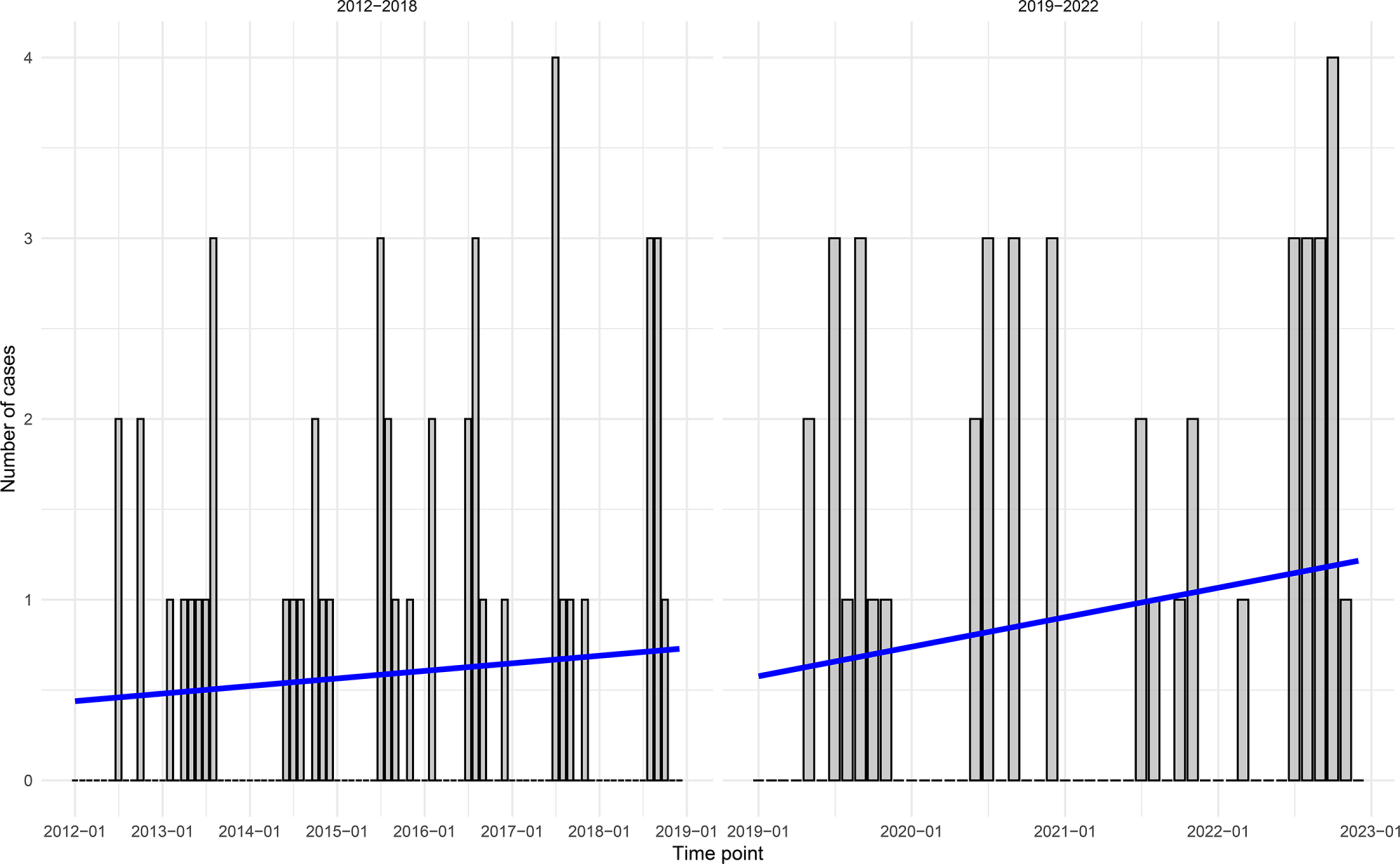
Temporal patterns of non-toxigenic cholera incidence, in Spain, 2012–2022. Case numbers are in bars and the fitted trend in blue. The panel on the left shows the cases from 2012 to 2019 and the panel on the right the cases from 2019 to 2022.

As observed in other studies, the number of NCV cases followed a periodic trend, with more cases in the summer and early fall. We also observed a significant increase in the number of isolates received after 2019 (Fig. 1; average 7 versus 11 cases/year; *P*-value=0.04). While the proportion of cases from both sexes was similar (58% were male), the probability of being a case increased with age (median age was 57 years old (yo), *χ*^2^ *P*-value=0.003, Fig. S1). Hospitalization status was unknown for 29 (31%) patients. Around 31% of the cases were hospitalized, and this proportion remained stable since 2017. However, we found that the probability of hospitalization increased with age (*P*-value=0.03). Information on symptoms was unavailable for 56% of the cases. Twenty-three (22%) cases reported diarrhoea, seven (7%) were with fever and six (6%) with otitis. Less common symptoms included sepsis (three cases above 50 yo), necrotizing fasciitis (two cases, one above 70 yo and age was unknown for the other) and convulsions/encephalitis (one case under 1 yo). Over the study period, three deaths were reported, two in 2020 and one in 2022. Two of them were children under 1 year old and one case was an adult above 65 years old.

### Diversity of NVC strains and identification of infectious source

**Fig. 2.**
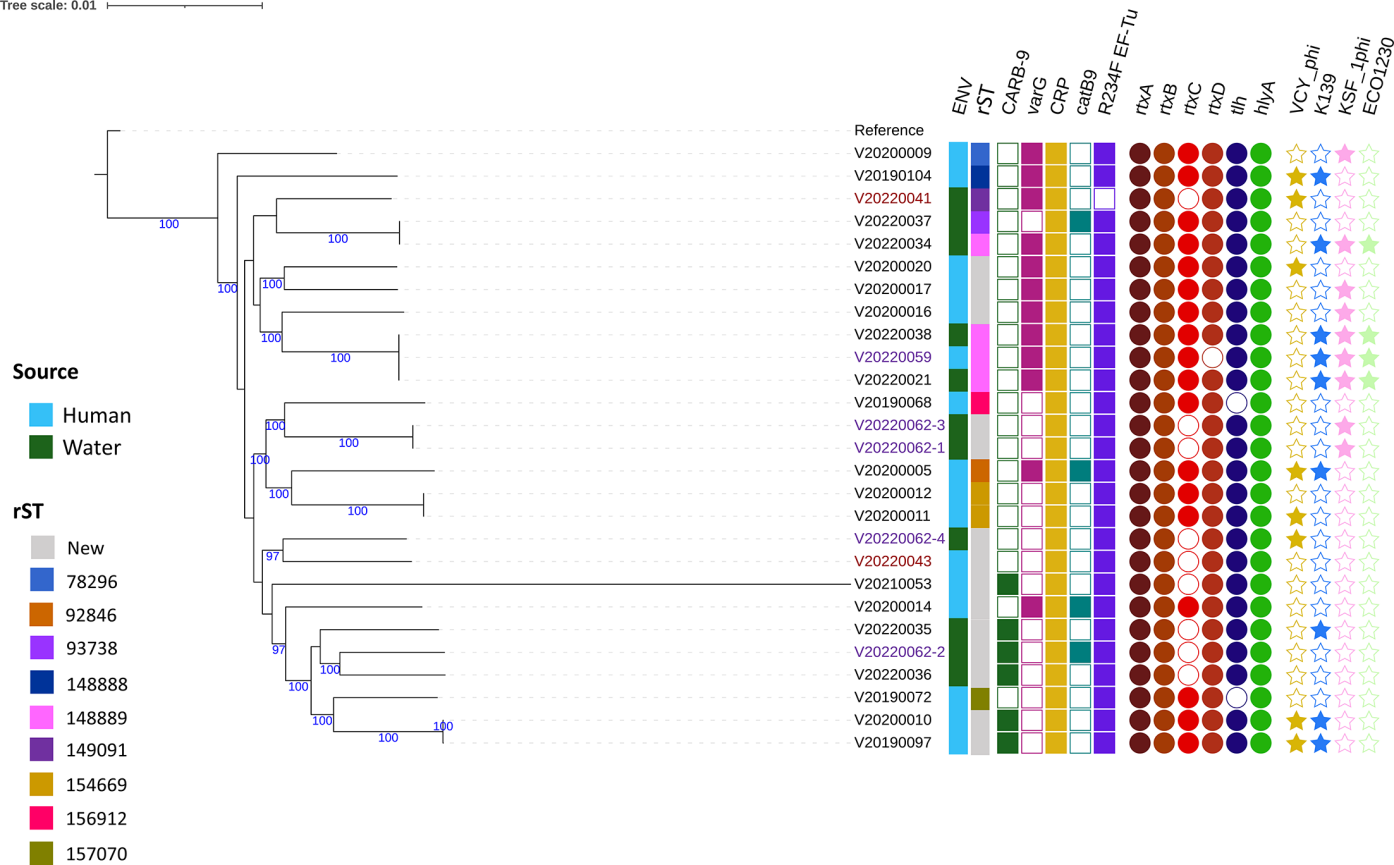
Maximum-likelihood phylogenetic tree of 27 non-toxigenic *V. cholerae* isolates. Sequences were aligned to genome LT907989 (chromosome 1) and LT907990 (chromosome 2). The following information is presented to the left of the isolate IDs: environment, rST, presence/absence of antimicrobial resistance genes (ARGs) (squares), virulence genes (circles) and prophages (stars). Bootstrap values >90 support are shown in the tree. Strains indicated in red and purple are related to the event of Melilla and Almeria, respectively.

Among the selected subset of 27 sequences from 2019 to 2022, both from environmental and human samples, we identified 13 new rST and 21 new ST in the dataset (Tables S1 and S2). By interrogating the PubMLST database for the identified STs, we observed that they were isolated in other countries in the past both in Europe and outside Europe (Table S2). Using the *V. cholerae* scheme of Enterobase, we performed a cgMLST analysis on human and environmental samples. The minimum spanning tree showed a high genetic diversity with only three clusters containing two human isolates, two environmental isolates and four isolates including a human isolate, respectively (Fig. S2). This was confirmed by the SNP analyses on human and environmental samples using Snippy with an average of 2125 SNPs between two strains (Fig. 2). In addition, we tested if genomic methods could be useful to identify the infectious sources of human cases. Therefore, we study two waterborne events where the infectious source was strongly established by previous epidemiological studies.

First, we study the relationship between a human case from Melilla in 2022 and a water sample. While the case tested positive, all the other inhabitants of the house tested negative using PCR. The parents of the case reported using water from an open-air water tank for dishwashing including feeding bottles and pacifiers. An environmental sample of the family water tank was taken and found to be positive by PCR. Because of the proximity of the open-air water tank to the sea, it was hypothesized that contamination occurred through marine birds’ faeces. The two strains were analysed by WGS and cgMLST analyses, and we found 1037 AD between the two strains.

Second, after the death of an NVC case in Almeria in 2022, an environmental study was conducted, and the sample was taken from the water tank used for showering. This time we realized four independent DNA extractions of the water samples. We found a diverse population in the water with at least three different strains, of which none was similar to the one that infected the case.

### Analysis of the resistome

All sequenced samples harboured the regulator of multidrug efflux gene *CRP* and the genes *almE* and *almF*, which can confer resistance to polymyxin and a mutation in the gene *parE* (D476N). Twenty-six (97%) strains carried the gene *almG* and a mutation in the prolongation factor *EF-Tu*, which has been associated with elfamycin resistance. The *bla_CARB-9_* gene, responsible for *β*-lactamase resistance, was identified in 21% of the samples, and resistance was confirmed phenotypically using disc-diffusion methods. The gene *varG*, also associated with resistance to *β*-lactamases, was found in 43% of the samples but was not associated with phenotypic resistance. Regarding resistance to chloramphenicol, the gene *cat*B9 was found in 12% of the strains, but phenotypical resistance was not observed. There was no difference between clinical and environmental samples regarding the presence of ARGs.

Two to four genes from the repeat in toxin (RTX) toxin cluster were present in all samples – *rtxA* (100%), *rtxB* (100%), *rtxC* (64%) and *rtxD* (96%). The pore-forming cytolysin gene *hlyA* and the thermolabile haemolysin gene *tlh* were present in 100 and 93% of the samples, respectively. No isolate was positive for accessory cholera enterotoxin (*ace*), zonula occludens toxin (*zot*) or toxigenic *V. cholerae* (*ctx*). We did not observe an association between the severity of the case and the presence of specific virulence factors. Similarly, the profile for virulence genes was mostly similar between clinical and environmental samples, with the exception of the gene *rtxC*, which was present in 82% of clinical isolates and only in 36% of environmental isolates.

Lastly, we detected between one and four prophages from the isolates but no plasmid replicons. The most frequent phage was the K139 (in 35% of the isolates) [22]. We also found the helper phage KSF-1φ in 32% of the isolates, the VCYφ in 28% of the isolates and a lytic *Escherichia coli* phage vB_EcoM_ECO1230 in 14 % of the isolates.

## Discussion

Here, we conducted a retrospective study and described the epidemiology and the genetic characteristics of NVC isolates collected in Spain from 2012 to 2022. We found that there was an increase in the number of cases sent to the NCM since 2019. However, this finding has two major limitations. First, samples are received on a voluntary basis, and therefore, we are probably underestimating the real incidence of NVC at the national level and the scale of the increase in vibriosis cases. Second, as the declaration of NVC cases is not mandatory in Spain, we cannot compare our data to an epidemiological database to establish the representability of our data. Nevertheless, the protocol for toxigenic *V. cholerae* O1/O139 surveillance includes a recommendation that the absence of toxin should be confirmed for all suspected cases since 2015 [23]. Thus, we believe that our data provide a good overview of the situation in Spain. In addition, the change in policy was not concomitant with the observed change in tendency, suggesting that the increase in cases might be epidemiologically relevant. The increase in cases since 2019 could be related to the increase in temperatures in mainland Spain (https://climateknowledgeportal.worldbank.org/country/spain/trends-variability-historical), seawater (https://www.ceam.eshttps://www.ceam.es/ceamet/SST/SST-trend.html) and natural water sources. Indeed, the link between heatwaves, leading to an increase in sea surface temperature, and vibriosis incidence has already been observed [24,25].

The isolates from most severe cases harboured the same virulence genes as other cases. From the pathogenicity factors, the genes *hlyA* and *rtxA* were detected in all samples, suggesting that the difference in severity was not associated with the characteristics of the strains. However, we identified age as a potential risk factor for infection and severity. Similarly, a study in Northern Europe also reported an increase in the number of cases with age for other *Vibrio* species but not for NVC [5]. We hypothesize that the correlation between infectious risk, severity and age might be related to the immunocompromised status or the underlying conditions among elderly people. Nevertheless, this observation would need to be confirmed with a larger dataset.

The resistance and virulence genes were very similar between the clinical and environmental strains as observed in a German study [15]. All the samples harboured at least two of the four *rtx* genes. The *rtx* gene cluster in *V. cholerae* encodes the presumptive cytotoxin (*rtxA*), an acyltransferase (*rtxC*), and an associated ATP-binding cassette transporter system (*rtxB* and *rtxD*, two proteins for toxin transportation) [26]. Phenotypically, these genes are proven to be associated with cytotoxicity by inducing apoptotic death [27,28]. We observed that a higher percentage of clinical samples on average harboured the gene *rtxC* compared to environmental isolates. Even though the *rtxC* gene has been described as an activator of the RTX toxin, its role in infection is unclear and the gene product is not necessary for the toxin function in an animal model [29]. Therefore, the gene’s absence in environmental samples might not have a functional impact, especially as the function of *rtxC* might be redundant with other genes of the *rtx* complex. In addition, we detected the presence of the K139 phage in one-third of Spanish isolates. Originally isolated from the serogroup O139, it is frequently detected in *V. cholerae* strains of serogroup O1 biotype El Tor. Previously, highly K139-related phage sequences were also detected in non-O1 and non-O139 strains, confirming our results [22]. A recent study suggested that K139 plays an ecological role by providing a competitive advantage for lysogenic strains in mixed populations containing non-lysogens [30]. This might be particularly important in a natural environment with a high diversity of strains. Finally, we also detect the KSF-1φ phage, which has been shown to promote *V. cholerae’s* acquisition of critical genes, altering its virulence or adaptation to its environmental niche [31].

Our MLST analysis showed a genetic heterogeneity between clinical NCV isolates, as the majority belonged to rSTs and STs not yet assigned in the PubMLST database. The same NCV STs were detected in other countries. This observation might result from common exposure to contaminated seafood or environmental spread of clones through, e.g. sea currents [32], plastic pollutants [33], ship ballast water [34] or waterbirds [35]. Similarly, the cgMLST analysis showed a high diversity of NVC, and we did not detect aggrupation of human cases at the national level. This could be explained if the sources of the infection were rapidly controlled or because of the low sensitivity of the surveillance system. WGS coupled with cgMLST and SNP analyses were insufficient to confirm the infectious source even in situations where epidemiological evidence was clear. It is important to note the limitations of the genomic methods in NVC surveillance. First, the diversity in water samples suggests that as many colonies as possible should be studied to establish such a link between environmental samples and human isolates. Thus, the colonies matching the clinical sample might have been missed. Similarly, patients could have been infected with multiple strains but only one was isolated and sequenced. Second, the timeliness of environmental sampling as well as the sampling methods might affect the representability of the samples. In this context, it is challenging to reject environmental exposure as the infectious source even if the isolated strains are discordant. Thus, control measures for waterborne outbreak should rely primarily on the epidemiological study and PCR results, even considering the limitations of these methods.

NCV infections are usually self-limiting and antibiotics are not administrated to patients [36]. However, when necessary, doxycycline is the first-line drug followed by azithromycin or ciprofloxacin. In our study, isolates were mostly pan-sensible apart from a small percentage of isolates resistant to *β*-lactamases. Therefore, it suggests that antimicrobial resistance in NVC should not necessarily raise public health concerns in Spain. However, the situation should be continuously monitored as we found virulence genes and phages previously described in *V. cholera* [31].

## supplementary material

10.1099/mgen.0.001315 Uncited Supplementary Material 1.

## Abbreviations

ace: accessory cholera enterotoxin
cgSNPs: core genome SNPs
ctx: toxigenic *V. cholerae*
MLST: multilocus sequence typing
NCM: National Center of Microbiology
NVC: non-toxigenic non-O1/non-O139 *Vibrio cholerae*
RTX: repeat in toxin
ST: sequence type
WGS: whole-genome sequencing
yo: years old
zot: zonula occludens toxin
